# Corticotomy Depth as a Modulator of Orthodontic Tooth Movement and PDL Stress—A Finite Element Study

**DOI:** 10.3390/ma18235290

**Published:** 2025-11-24

**Authors:** Anna Ewa Kuc, Kamil Sybilski, Jacek Kotuła, Grzegorz Hajduk, Magdalena Sulewska, Szymon Saternus, Justyna Ewa Kulikowska-Kulesza, Małgorzata Kotarska, Beata Kawala, Jerzy Małachowski, Michał Sarul

**Affiliations:** 1 Department of Dentofacial Orthopedics and Orthodontics, Wroclaw Medical University, Krakowska 26, 50-425 Wroclaw, Poland; 2 Faculty of Mechanical Engineering, Military University of Technology, ul. gen. Sylwestra Kaliskiego 2, 00-908 Warsaw, Poland; 3 Chair and Department of Oral Surgery, Medical University of Lublin, Doktora Witolda Chodźki 6 Street, 20-093 Lublin, Poland; 4 Department of Periodontal and Oral Mucosa Diseases, Medical University of Białystok, ul. Waszyngtona 13, 15-269 Bialystok, Poland; 5 Faculty of Law, University of Bialystok, Mickiewicza 1, 15-213 Bialystok, Poland; 6 Department of Integrated Dentistry, Wroclaw Medical University, 50-425 Wroclaw, Poland

**Keywords:** corticotomy, orthodontic expansion, finite element analysis, periodontal ligament, bone density, tooth movement, regional Acceleratory phenomenon (RAP), PDL stress distribution, regulatory framework

## Abstract

**Introduction:** The aim of this study was to evaluate the effect of corticotomy incision depth on tooth movement and stress distribution in the periodontal ligament (PDL) during orthodontic expansion using finite element analysis (FEA). The demand for accelerated and biologically safe orthodontic techniques has highlighted the importance of understanding biomechanical responses to surgical adjuncts like corticotomy. **Objective**: The aim of this study is to assess the effect of corticotomy depth on tooth movement and periodontal ligament stress distribution during orthodontic treatment using finite element analysis. **Materials and methods:** A 3D FEM model was developed based on CBCT and intraoral scans to replicate anatomical structures and simulate clinical orthodontic scenarios. Four conditions were analyzed: no corticotomy and corticotomy incisions of 1 mm, 2 mm, and 3 mm depths, applied between roots and above the apex region. Different cortical bone densities were tested using Young’s modulus values (12,500 MPa–27,500 MPa). Stress and displacement values were measured in both the crown and root regions. **Results:** The 3 mm corticotomy, penetrating through the cortical plate into the cancellous bone, significantly increased crown displacement (up to 26% in low-density bone) and altered root tipping patterns, reducing root movement relative to the crown. Shallower incisions (1–2 mm) had minimal effects. Despite increased movement, stress concentration in the cervical PDL region remained high across all scenarios, particularly in the premolar area, exceeding the 4.7 kPa threshold associated with tissue ischemia. **Conclusions:** Corticotomy depth is a critical factor for optimizing orthodontic tooth movement. Penetration into cancellous bone (3 mm) appears necessary to induce both: not only the Regional Acceleratory Phenomenon (RAP) but also to enhance displacement. However, this approach does not significantly reduce cervical PDL stress and offers limited periodontal protection. Individual planning based on bone density, morphology, and anatomical limitations is essential for balancing treatment efficiency and periodontal safety.

## 1. Introduction

In recent years, there has been a significant increase in the demand for orthodontic treatment, driven by both functional needs and increasing aesthetic awareness among patients. However, the choice of an appropriate therapeutic strategy, particularly the decision between treatment involving premolar extractions and non-extraction approaches, remains a subject of debate among orthodontic specialists. Research indicates that premolar extraction may facilitate better space management and occlusal stability, but concerns have been raised regarding its impact on temporomandibular joint health and facial profile deterioration [1,2,3,4,5,6,7,8].

One of the primary proponents of extraction was Charles H. Tweed, who in the 1940s introduced the Tweed Technique. This approach relied on the extraction of four first premolars to achieve stable, functional, and aesthetically pleasing occlusion. Tweed argued that extractions could eliminate anterior tooth protrusion, align incisors to enhance facial profile aesthetics, and prevent relapse and instability. The extraction approach gained widespread popularity, with the prevailing view that stable Class I occlusion could not be achieved without extractions. However, in the 1980s and 1990s, controversies emerged regarding the impact of premolar extractions on facial profile, including excessive lip retrusion and a premature ageing effect, as well as concerns about occlusal instability, relapse of crowding despite extractions, and adverse effects on airway function and speech. During this period, reports also suggested an increased risk of root resorption associated with extractions. These developments sparked growing interest in non-extraction techniques, such as arch expansion, interproximal reduction—also known as ‘stripping’, or the use of temporary anchorage devices (TADs) [9,10,11,12]. Non-extraction treatments, which often involve distalisation and arch expansion, may increase the risk of alveolar bone dehiscence and fenestration, particularly in patients with a thin gingival biotype or fragile bone morphology. Such structural changes may predispose patients to gingival recession, underscoring the need for individualised risk assessment prior to initiating orthodontic treatment [13,14,15,16,17,18,19,20,21,22]. Currently, the decision to extract first premolars is made more selectively and on a case-by-case basis. However, arch expansion may still be necessary, regardless of the extraction decision.

The biological mechanism of tooth movement relies on the periodontal tissues’ response to applied mechanical forces. In areas of compression, reduced blood flow in periodontal vessels and the displacement of periodontal ligament (PDL) fluid into bone canaliculi activate molecular pathways, such as the RANKL/OPG signalling cascade, leading to osteoclast activation and bone resorption. Conversely, in areas of tension, increased blood flow, fluid influx into the PDL space, and activation of osteoblast production promote bone formation. These processes are mediated by PDL cells, which respond to mechanical stimuli through activation of signalling pathways, such as RANKL/OPG, facilitating bone remodelling [23,24,25,26,27,28]. In vivo studies confirm that orthodontic forces upregulate RANKL expression in PDL fibroblasts and osteoblasts, with an increased presence of TRAP-positive cells (osteoclasts) within 24–72 h of force application. Animal model studies have demonstrated that blocking TNF-α inhibits bone resorption induced by orthodontic forces [29,30]. Prostaglandins, particularly PGE2, are produced by PDL and endothelial cells in response to mechanical pressure, upregulating RANKL and IL-1β expression. Together with pro-inflammatory cytokines IL-1β and IL-6, these enhance osteoclastogenesis on the compression side. In contrast, TGF-β (Transforming Growth Factor-β) may exert an anti-resorptive effect and support osteoblast differentiation on the tension side [31,32,33,34]. While these processes are desirable during tooth movement along the alveolar ridge, resorption of the buccal cortical plate during expansion can lead to undesirable outcomes, such as bone dehiscence, fenestration, and gingival recession [35,36].

For many years, corticotomy has been employed to leverage the Regional Acceleratory Phenomenon (RAP), a biological process involving accelerated remodelling of bone and surrounding tissues in response to surgical trauma or mechanical irritation. This procedure aims both to expedite orthodontic movement and protect periodontal health. However, its efficacy in the aspect of protecting periodontal health remains controversial due to the limited quality and heterogeneity of scientific studies and clinical protocols [37,38,39,40,41]. Understanding the biomechanics of tooth movement, including stress distribution in the PDL and the impact of various forces and adjunctive procedures, is critical for optimising orthodontic treatment. The finite element method (FEM) is a valuable tool for analysing these processes, enabling the simulation of various therapeutic scenarios and their potential effects on periodontal tissues [42,43]. The FEM study omitted the clinically studied RAP phenomenon affecting the acceleration of tooth movement and focused on the influence of corticotomy on the loading of the periodontium, which influences the process of remodeling and bone resorption during expansion.

In light of these considerations, this study aims to evaluate the influence of corticotomy depth on tooth movement and stress distribution in the periodontal ligament (PDL) during orthodontic treatment, using finite element analysis (FEM). The objective is to provide data that may assist clinicians in making informed therapeutic decisions, minimising the risk of periodontal complications, and enhancing the efficiency and stability of treatment outcomes.

## 2. Materials and Methods

To achieve the objective of the study, a research methodology based on the finite element method was used. This methodology allows for multiple studies to be conducted under exactly the same conditions. This makes it possible to accurately verify the impact of selected treatment parameters and patient conditions, e.g., on the state of stress and displacement in the stomatognathic system.

The numerical model used for the described research was constructed using two bone imaging methods: computed tomography and 3D scanning [44,45]. A CBCT was performed for the selected patient, resulting in DICOM files representing internal structures in grayscale. Based on these files, layer by layer, the compact bone, cancellous bone, teeth, and empty spaces were manually outlined. MIMICS 18.0 software (Materialise, Leuven, Belgium) was used for this purpose [46], in which masks were defined for each outline by filling them with a uniform color. This created surfaces on each layer of the DICOM image representing a cross-section of a given structure. Thanks to the high accuracy of CBCT, the boundary between compact and cancellous bone was clearly visible. However, in reality, these two structures may have different density ranges and, consequently, different stiffness ranges. In the study described, it was assumed that the entire compact bone would have one stiffness and the entire cancellous bone would have another, significantly lower stiffness. The bone thicknesses obtained were additionally consulted with orthodontic and oral surgeons.

**Figure 1:**
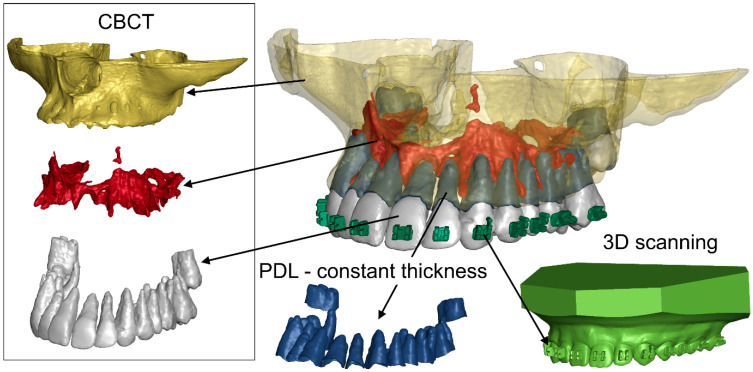
Geometries of structures reproduced in the FEM model.

After creating masks for each layer, a 3D model of the structures was determined. This process is carried out by extracting cross-sections of a given structure located on an adjacent layer. Therefore, the smaller the distance between the layers of the DICOM image, the more accurate the 3D model. The results of this process were saved in STL files (Figure 1).

CBCT imaging does not accurately reproduce enamel. Therefore, the geometry of the teeth and brackets was additionally determined using 3D intraoral scanning. It was also difficult to accurately determine the geometry of the PDL based on CBCT. Therefore, in accordance with the literature, a constant PDL thickness of 0.25 mm was assumed in the numerical model [47].

**Figure 2:**
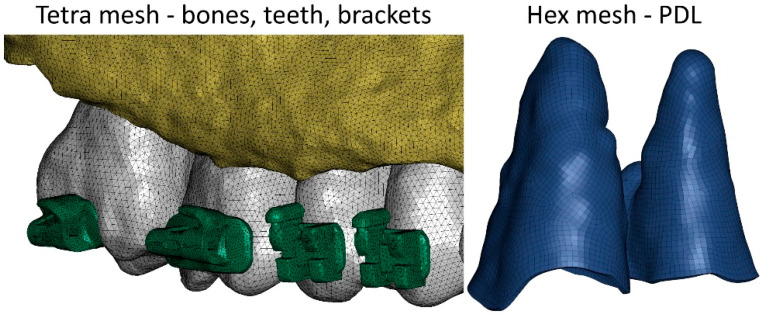
Finite element mesh used in the model.

Due to the large volume of the skeletal system reproduced in the numerical model, it was very important to select the appropriate type and size of finite elements. The aim of the study was to determine, among other things, the loads acting in the PDL; hence, these structures were modeled using hexagonal elements. The mesh size was set to 0.25 mm. Other structures, such as bones, teeth, and brackets, were modeled using tetragonal elements. The average side length of the element was 0.3 mm (Figure 2). In total, the numerical model was constructed using over 4.5 million elements and over 1.2 million nodes. The entire mesh was meshed using Hypermesh 2023 (Altair, Troy, MI, USA).

**Figure 3:**
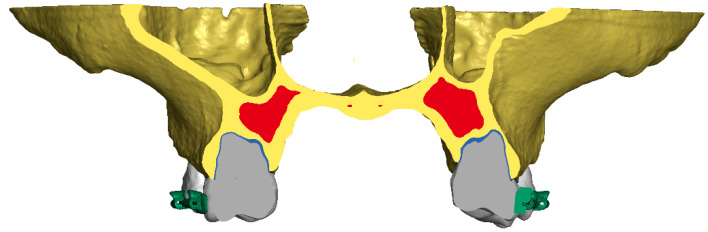
Cross-section showing the representation of internal structures in the model.

The combination of all structures allowed for the reproduction of the complete structure of the patient’s skull without corticotomic incisions. Individual structures were connected either by sharing nodes or by gluing using TIED (LS-Dyna) contact (Figure 3).

**Figure 4:**
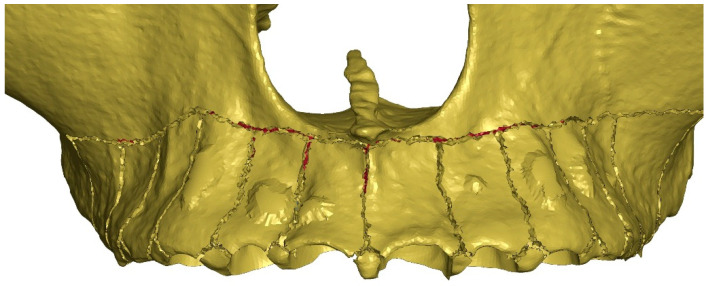
Corticotomy incision pattern (G3 = 3 mm).

As part of the study, four clinical scenarios were analyzed, which differed in the depth of corticotomy incisions (G0 = 0 mm; G1 = 1 mm; G2 = 2 mm; G3 = 3 mm). For each scenario, six additional compact bone stiffness values were analyzed (E = 12,500 MPa; 15,500 MPa; 18,500 MPa; 21,500 MPa; 24,500 MPa; 27,500 MPa), giving a total of 24 variants. The incisions were introduced into the numerical model by removing elements to the appropriate depth according to the geometric pattern shown in the figure below (Figure 4). The incisions were made on the buccal side. The extension of the osteotomy was determined by the mesio-distal dimension of the tooth roots as well as by the position of the apices of the roots. The vertical cuts ended 5 mm apically from the crest and then Y-shape spread towards the neighboring teeth. The horizontal corticotomy was performed approximately 2–4 mm apically above the root apices. The depth of the incisions was limited by the thickness of the cortical plate at the maxillary sinus. Where cancellous bone was anatomically present, incisions were made to a depth of 1, 2, and 3 mm, respectively.

**Figure 5:**
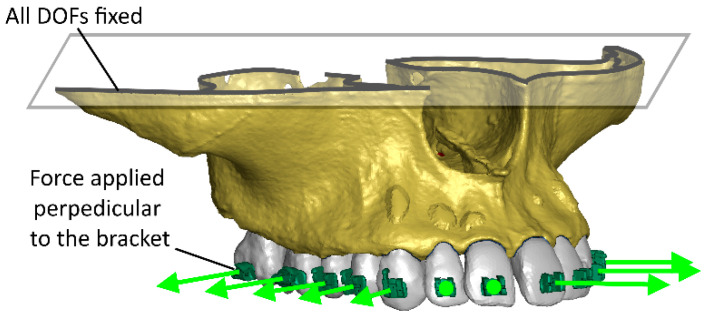
Initial and boundary conditions in the FEM model.

In each case, a conventional orthodontic expansion force of 100 g (F = 1 N) per lateral tooth was applied. To standardize the mechanical conditions across different bone stiffness levels, a simplified 1 N transverse load was applied instead of modeling the full archwire–bracket contact. This approach isolates the influence of corticotomy depth while avoiding additional variables such as wire stiffness, friction, or slot clearance. The authors acknowledge this as a simplification and plan to extend future models to include the full wire–bracket interaction for greater biomechanical realism. The fixation was defined by removing all degrees of freedom from the elements located on the upper surface of the model (Figure 5).

In the numerical model, it was verified that the brackets, teeth, and bones would be subjected to low loads and that there would be no significant stresses in these components. It was therefore assumed that these structures would be modeled using an isotropic, linear elastic constitutive model. The brackets were made of steel. The material data for the above components are given in Table 1.

During the research, the greatest emphasis was placed on modeling the behavior of the periodontal ligament (PDL). Ogden’s hyperelastic model was used to describe its strength properties. This model describes the behavior of the material using the strain energy function:

(1) $$W=\sum_{i=1}^{3}\sum_{j=1}^{n}\frac{\mu_{j}}{\alpha_{j}}\left(\lambda_{i}^{\alpha }j-1\right)+K\left(J-1-\ln J\right)$$

where *W* is the strain energy potential, *λ_i_* is the main deviant stretches, *µ_i_* and *α_i_* are material parameters, *J* is the determinant of the elastic strain gradient, and *K* is the volume modulus. The bulk modulus is calculated using the values of Poisson’s ratio and Young’s Modulus. The parameters presented in Table 2 [50] were used to describe the behavior of the PDL.

Displacement was measured at the mesiobuccal cusp centroid (crown) and at the root apex node set (root) of the first premolar (tooth 15), which represents the main site of expansion load transfer. The pressure values in Table 3 correspond to the maximum hydrostatic pressure (positive values indicate compression, negative values indicate tension) within the PDL for the same tooth. Clarification has been added to Table 3 to specify these reference points.

## 3. Results

Upon application of a 100 g expansive force to the maxillary posterior teeth in the finite element method (FEM) model without corticotomy, the stresses in the periodontal ligament (PDL) and the displacements of the crown and root vary depending on bone density (Table 3). However, the highest stress values are consistently located in the marginal periodontal region.

**Figure 6:**
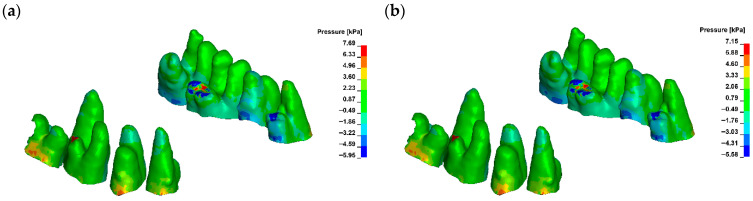
Maximal hydrostatic pressure in PDL without corticotomy G0 ((**a**) E = 12,500, (**b**) E = 27,500).

**Figure 7:**
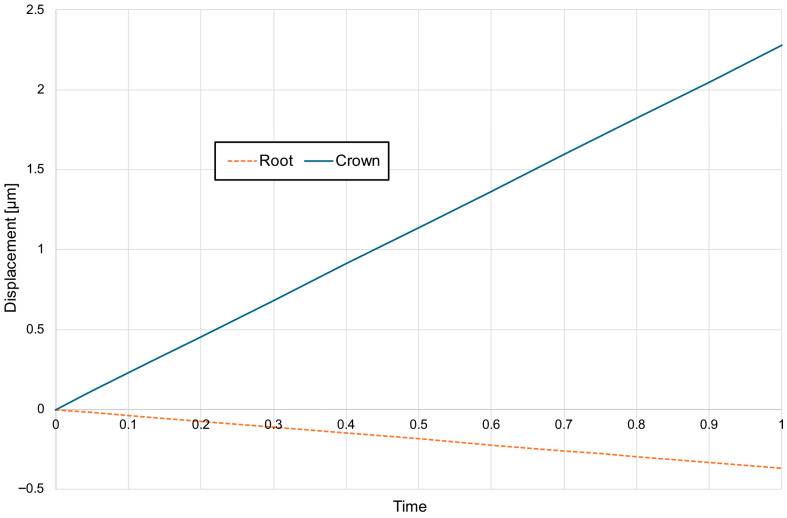
Chart of displacement of the crown—blue and the root—orange without corticotomy G0.

**Figure 8:**
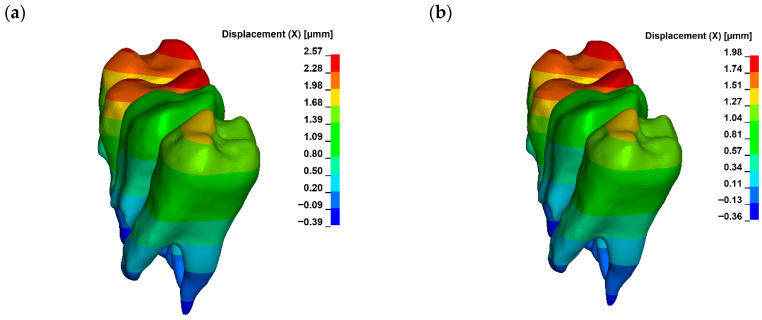
Displacement without corticotomy G0 ((**a**) E = 12,500, (**b**) E = 27,500).

For a bone modulus of elasticity (E) of 12,500 MPa, the maximum compressive stresses reach approximately 7.69 kPa, with a crown displacement of 2.57 μm. At a higher E of 27,500 MPa, the stresses are slightly lower at 7.15 kPa, and the crown displacement is reduced to 1.98 μm. The observed movement is characterised as uncontrolled tipping, where the root displaces in the opposite direction to the crown, with the root displacement being approximately five times smaller than that of the crown (Figure 6, Figure 7 and Figure 8).

A corticotomy incision performed according to the protocol with a depth of 1 mm results in a minimal increase in tooth displacement of approximately 2%, without significantly affecting the stress distribution in the periodontal ligament (PDL) (Table 3).

A corticotomy incision of 2 mm increases the movement of the crown by about 12% in dense cortical bone and 8% in low-density cortical bone. The maximal hydrostatic pressure increases by about 3% while the minimum hydrostatic pressure is the same (Table 3)

**Figure 9:**
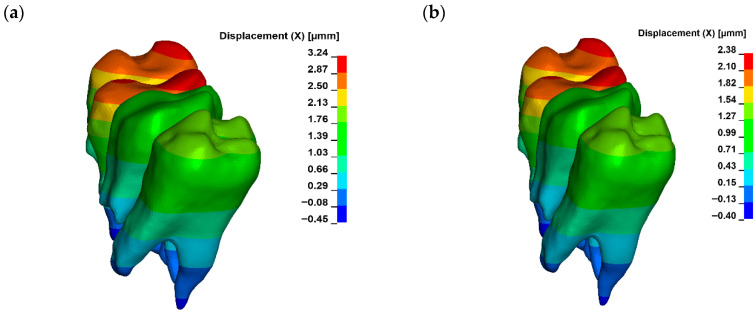
Displacement with corticotomy 3 mm G3 ((**a**) E = 12,500, (**b**) E = 27,500).

**Figure 10:**
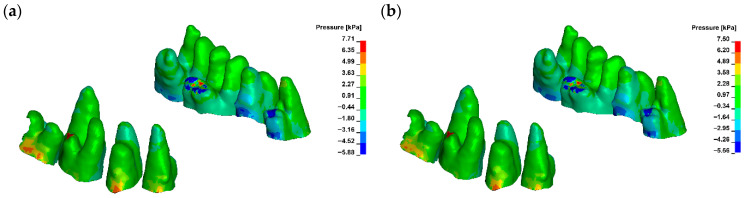
Maximal hydrostatic pressure in PDL corticotomy 3 mm G3 ((**a**) E = 12,500, (**b**) E = 27,500).

**Figure 11:**
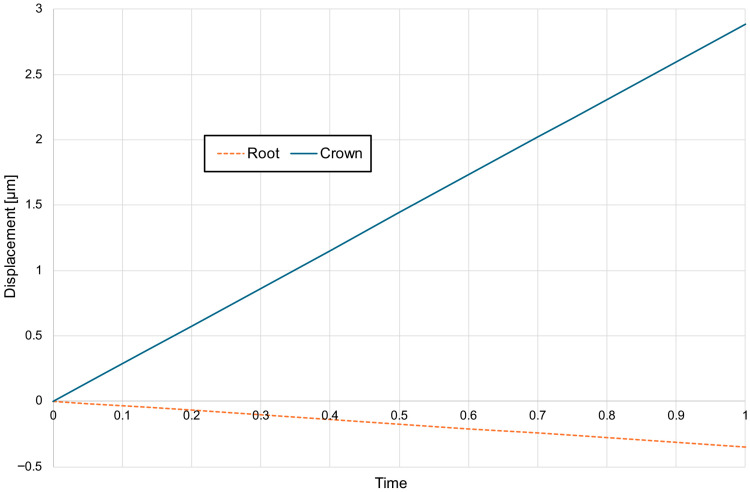
Chart of displacement of the crown—blue and the root—orange with 3 mm corticotomy G3 E = 12,500.

A corticotomy incision of 3 mm, which consistently penetrates the entire thickness of the cortical plate and reaches the cancellous bone, induces a significant change in the magnitude of displacement for both the crown and the root. For a bone modulus of elasticity (E) of 12,500 MPa, the displacement is 3.24 μm (26%), while for E = 27,500 MPa, it increases to 2.38 μm (20%) (Figure 9). The hydrostatic pressure values are similar and the change is clinically not significant (Figure 10). This also alters the extent of uncontrolled tipping, with the crown displacing 12 times more than the root (Figure 11). The root continues to move in the opposite direction to the crown, but the magnitude of root displacement is approximately two times smaller than that observed without corticotomy.

## 4. Discussion

The paper is a continuation of a series of publications on the assessment of loads occurring in the stomatognathic system [45,46]. Compared to previous works, the scientific and novel aspect of this paper involves the development of a series of numerical models that take into account different depths of corticotomy incisions and the stiffness of compact bone. The developed models were used to determine the impact of the above parameters on tooth displacement and PDL loads.

The results of this study indicate a significant increase in tooth movement after a 3-mm corticotomy, without a significant reduction in periodontal ligament (PDL) loading. These results are consistent with previous biomechanical studies that have shown that deeper corticotomies primarily increase tooth movement rather than reduce PDL loading. Although these studies primarily focus on other orthodontic movements, their results are comparable.

In a clinical study by Alighani et al. [51], micro-osteoperforations (MOPs) with a depth of 2–3 mm were employed to accelerate canine retraction. The study found that MOPs increased the rate of canine retraction by 2.3 times compared to the control group, suggesting that penetration into cancellous bone significantly enhances the efficiency of orthodontic treatment [52].

Similarly, in a finite element method (FEM) analysis by Uysal et al., the impact of corticotomy combined with zygomatic anchorage on maxillary molar intrusion was evaluated. The results indicated that a corticotomy depth of approximately 3 mm significantly reduced PDL stresses, potentially facilitating more effective tooth movement [53]. In contrast, a three-dimensional FEM analysis by Pacheco et al. comparing different corticotomy techniques during canine retraction found that the format of the corticotomy had minimal impact on PDL stress distribution, suggesting that depth and location may be more critical than the specific corticotomy pattern [54]. Animal studies have shown that tooth displacement increases significantly with greater numbers or depths of alveolar bone perforations. Both corticotomy (incision of the cortical plate) and osteotomy (complete transection of the cortical plate into cancellous bone) effectively accelerate tooth movement by promoting bone remodelling. Bone remodelling was faster and completed earlier following osteotomy compared to corticotomy, resulting in accelerated tooth movement in the early stages of orthodontic treatment [55].

The results of the present study indicate that only a corticotomy depth exceeding the cortical plate and reaching the cancellous bone (approximately 3 mm) significantly enhances tooth displacement, supporting the hypothesis of activation of the Regional Acceleratory Phenomenon (RAP) but without the justified protection mechanism on marginal periodontium because of no significant changes in PDL hydrostatic pressure. However, this raises questions about the impact of such interventions on periodontal tissue health and cortical bone integrity, particularly in the context of orthodontic expansion [55,56,57].

The literature reveals inconsistencies regarding the long-term safety of corticotomy. According to a recent systematic review by Kuc et al. [40], the effectiveness of corticotomy remains controversial. De Stefani et al. [58] emphasised that corticotomy should be limited to cases of moderate expansion and always accompanied by bone augmentation to ensure comprehensive root support. However, the high risk of systematic bias in their analysis limits the evidential value of these conclusions [40,58]. Dab et al. [59] noted that, despite low-level evidence of no adverse effects, a moderate increase in bone density (approximately 7%) can be expected following augmentation. Similar effects—increased bone density and alveolar ridge thickness—were reported by Gao et al. [60] in studies involving Periodontally Accelerated Osteogenic Orthodontics (PAOO) with additional bone augmentation, although the quality of the analysed studies was suboptimal. Apalimova et al. [61] compared various surgically assisted orthodontic techniques, concluding that classic corticotomy (with flap elevation) is more effective than piezocision, albeit more invasive. Notably, they found no significant differences in complications such as root resorption, gingival recession, or pulp vitality loss compared to conventional treatment. Regarding soft tissues, Al-Ibrahim et al. [62] demonstrated that piezocision combined with self-ligating appliances does not cause gingival recession, and the use of low-frequency vibrations or corticotomy does not adversely affect periodontal parameters. The moderate risk of systematic bias in their study lends credibility to these findings. Furthermore, Kamal et al. [63] and Alsino et al. [64] suggest that PAOO may not only accelerate treatment but also improve bone conditions and periodontal tissue stability by increasing alveolar ridge thickness. Wang et al. [65] reported that thickening the gingival biotype with soft tissue augmentation can provide clinical benefits, particularly in patients with a thin gingival biotype and unfavourable bone morphology.

Conversely, studies evaluating cortical bone changes following piezocision-assisted upper arch expansion found no significant adverse changes in the cortical plate, except in the premolar region, where a reduction in buccal cortical plate thickness was observed. However, due to the case series design and low evidential value, these findings carry a high risk of systematic bias. The reduction in cortical plate thickness in the premolar region aligns with the present study’s results, as the highest PDL stresses accumulate in this area, leading to bone resorption [66]. Corticotomy may result in minimal periodontal tissue changes, with outcomes depending on the technique used (e.g., incision method and depth). It is generally recommended to perform corticotomy incisions 3 mm below the bone crest and 3–5 mm away from root apices. Proper surgical management during flap preparation is also critical.

Sulewska et al. [67] highlighted this aspect, performing incisions that preserve interdental papillae in accordance with minimally invasive surgical principles to protect marginal periodontal tissues. Long-term observations (over 5 years) by the same research team [68] found no adverse effects of corticotomy-assisted orthodontic arch expansion on periodontal health in terms of soft tissue, focusing on the soft tissue aspect. However, the authors did not address incision depth, which could aid in interpreting the present study’s findings [67,68]. These results may explain the variability in research outcomes due to the lack of clear guidelines on incision depth. Comparing the effectiveness of a 1 mm corticotomy, which results in only a 2% change in displacement, to deeper 3 mm corticotomies, which produce approximately 40% greater displacement, without considering bone density—a critical factor—may lead to erroneous and controversial conclusions.

The differentiation of the periodontal ligament load after corticotomy depending on the initial bone density seems particularly interesting. This opens up further discussion regarding the potential reduction of the risk of root apical resorption and the risk of dehiscence. The study by Kuc et al. [45] showed that during incisor retraction, low bone density predisposes to a greater response and stress in the periodontal ligament, which significantly increases the risk of root resorption in patients with, e.g., high bone density or osteoporosis. While in the current study, on the contrary, a denser vestibular plate causes greater stress during expansion in the marginal periodontium and increases the risk of hyalinization and bone and soft tissue destruction. Detailed studies in this aspect are indicated to be able to draw deeper conclusions and clinical guidelines.

During expansion, PDL stresses may be particularly significant for periodontal tissue protection. Across all corticotomy depths, the cervical region, especially around premolars, is highly susceptible to undermining resorption due to stresses exceeding the 4.7 kPa threshold, which occludes blood vessels. This may lead to hyalinisation, tissue necrosis, and increased bone resorption, potentially causing bone dehiscence and gingival recession. These effects are linked to osteoclast and cementoclast activation in response to tissue ischaemia caused by prolonged PDL overloading. The 20–40% variation in PDL stresses depending on bone density, coupled with their accumulation in the marginal periodontal region, suggests that corticotomy as a standalone procedure may not significantly reduce marginal bone resorption compared to non-corticotomy cases. However, when performed, corticotomy should be deep enough to maximise positive outcomes [55,69,70,71,72,73,74,75].

A beneficial aspect of corticotomy is the reduced root movement in the opposite direction to the crown compared to non-corticotomy cases. This may decrease the risk of root resorption by reducing stresses on the limited PDL surface surrounding the root apex. Increased crown displacement, and thus significantly accelerated orthodontic movement, is observed following deep corticotomy. Notably, the lack of clear visibility of cancellous bone structure in CBCT scans necessitates caution when planning corticotomy. In such cases, limiting incision depth and considering additional imaging or consultation with an experienced specialist is recommended. In certain scenarios, cancellous bone may be virtually absent between tooth roots, particularly in specific anatomical regions or patient populations. This is especially relevant in the anterior mandible, where histological and imaging studies indicate that the space between roots may consist entirely of cortical bone without evident trabeculation. A similar issue may arise in patients with significantly reduced alveolar ridge height (e.g., due to periodontal disease), where sclerotisation may reduce or eliminate the cancellous bone layer [76]. Wilcko et al. [77], pioneers of the PAOO technique, described cases where incisions were made without penetrating cancellous bone due to its near absence. Kim et al. [78] noted that CBCT imaging revealed minimal or absent cancellous bone in the anterior mandible in some patients. Melsen [79] highlighted that the bone structure in the anterior maxilla and mandible may be almost entirely cortical, significantly influencing orthodontic responses. The depth of corticotomy incisions in the molar region warrants individual consideration. Depending on the technique, superficial corticotomy incisions in the maxillary molar region are recommended to minimise the risk of maxillary sinus damage [80,81,82,83]. However, this may reduce the procedure’s effectiveness and limit the extent of tooth displacement and PDL stresses. Current evidence suggests that incisions should target only the cortical bone layer without penetrating deeply into the cancellous bone. Excessively deep incisions may lead to sinus perforation or mucosal damage, resulting in serious complications (e.g., infection). Therefore, the risks must be weighed, and digitally navigated corticotomy is advisable to optimise outcomes safely. Preoperative imaging is critical. Modern techniques such as computed tomography (CT) or panoramic radiographs allow precise assessment of the anatomical relationship between tooth roots and the maxillary sinus, enabling accurate determination of incision depth to minimise complications. Contemporary approaches emphasise tailoring the procedure to the patient’s anatomy. In cases where the maxillary sinus is shallow or close to the root apices, incisions should be more conservative to avoid perforation [80,81,82,83].

From a regulatory perspective, surgical ancillaries such as corticotomy and piezocision are considered medical procedures under the European Medical Devices Regulation (MDR 2017/745) when specialized piezoelectric instruments or systems are used [84]. The regulation’s enhanced clinical evidence requirements and post-market surveillance obligations create documentation standards for device-related complications. Although this finite element model does not assess risk, soft tissue response, or device classification, it provides quantitative biomechanical data that can support preclinical assessments required by the evidence-based MDR framework. In this simulation, penetration into cancellous bone appears to exacerbate tooth displacement, but its clinical application should be assessed on a case-by-case basis, depending on bone morphology and anatomical safety. Therefore, the numerical results should be interpreted as supplementary scientific evidence rather than direct clinical guidance.

Considering all factors influencing procedure efficacy and anatomical constraints, corticotomy incisions should reach the cancellous bone where feasible and safe to maximise therapeutic benefits.

Limitations: The study used a numerical model developed on the basis of CBCT and scans performed on a single patient. The model accurately reproduced the geometry of bone structures and teeth. PDL thickness was assumed based on the literature. It should be noted that this model has not been validated by experimental tests, which means that the results obtained using it cannot be used to accurately predict changes during treatment of this particular patient or other patients. Due to the lack of accurate material characteristics, the study analyzed different stiffnesses of compact bone. Nevertheless, there may be significant differences in bone structure and the ratio of compact to cancellous bone between different patients. For this reason, the results presented in this article should be treated as results showing the trend of displacement and hydrostatic pressure changes in the case of changes in bone stiffness and the depth of corticotomy incisions.

## 5. Conclusions

The findings of this study, which demonstrate a reduction in periodontal ligament (PDL) stresses and a greater extent of tooth displacement only after penetration into cancellous bone, provide a biological rationale for controlled, selective, and deeply but individually planned corticotomy to assist orthodontic treatment. Such corticotomy accelerates tooth movement but offers limited protective function for periodontal tissues—provided that the periodontal phenotype is assessed beforehand.

## Figures and Tables

**Table 1:** Material data used to describe the material behavior [44].

Component	Young’s Modulus [MPa]	Poisson’s Ratio [ ]
Steel	210,000	0.30
Tooth [48]	18,600	0.31
Cancellous [49]	2000	0.30

**Table 2:** Parameters used for describing the PDL [44].

*µ*_1_ [MPa]	*α*_1_ [MPa]	Poisson’s Ratio [ ]
2.5 × 10^−3^	150	0.46

**Table 3:** The values of displacement and minimum and maximum hydrostatic pressure depending on the bone density and depth of incision: G0—without corticotomy, G1—1 mm corticotomy, G2—2 mm corticotomy, G3—3 mm corticotomy.

Displacement [µm]						
	Young Modulus [MPa]					
	12,500	15,500	18,500	21,500	24,500	27,500
G0	2.57	2.37	2.23	2.13	2.04	1.98
G1	2.63	2.42	2.27	2.16	2.08	2.01
G2	2.89	2.64	2.47	2.34	2.24	2.15
G3	3.24	2.95	2.75	2.59	2.48	2.38
**Hydrostatic Pressure Min [kPa]**						
	**Young Modulus [MPa]**					
	**12,500**	**15,500**	**18,500**	**21,500**	**24,500**	**27,500**
G0	−5.95	−5.84	−5.76	−5.69	−5.63	−5.58
G1	−5.95	−5.85	−5.76	−5.69	−5.63	−5.58
G2	−5.95	−5.84	−5.76	−5.69	−5.63	−5.58
G3	−5.88	−5.79	−5.72	−5.66	−5.61	−5.56
**Hydrostatic Pressure Max [kPa]**						
	**Young Modulus [MPa]**					
	**12,500**	**15,500**	**18,500**	**21,500**	**24,500**	**27,500**
G0	7.69	7.53	7.4	7.3	7.22	7.15
G1	7.69	7.53	7.41	7.3	7.22	7.15
G2	7.94	7.77	7.64	7.54	7.45	7.38
G3	7.71	7.66	7.62	7.58	7.54	7.5

## Data Availability

The original contributions presented in this study are included in the article. Further inquiries can be directed to the corresponding authors.
